# Mechanisms of manipulation: a systematic review of the literature on immediate anatomical structural or positional changes in response to manually delivered high-velocity, low-amplitude spinal manipulation

**DOI:** 10.1186/s12998-024-00549-w

**Published:** 2024-09-11

**Authors:** Kenneth J. Young, Charlotte Leboeuf-Yde, Lindsay Gorrell, Cecilia Bergström, David W. Evans, Iben Axén, Kenneth Chance-Larsen, Olivier Gagey, Vasileios Georgopoulos, Guillaume Goncalves, Catherine Harris, Steen Harsted, Roger Kerry, Edward Lee, Christopher McCarthy, Casper Nim, Luana Nyirö, Petra Schweinhardt, Steven Vogel

**Affiliations:** 1 Allied Health Research Unit, University of Central Lancashire, Preston, UK; 2 Department of Regional Health Research, University of Southern Denmark, Odense, Denmark; 3 University of Central Lancashire, Preston, UK; 4 Department of Chiropractic Medicine, Integrative Spinal Research Group, Balgrist University Hospital, University of Zurich, Zurich, Switzerland; 5 Department of Clinical Sciences, Obstetrics and Gynecology, Umeå University, Umeå, Sweden; 6 School of Sport, Exercise and Rehabilitation Sciences, University of Birmingham, Edgbaston, Birmingham, UK; 7 Karolinska Institutet, Institute of Environmental Medicine, Nobels V. 13, 177 77 Stockholm, Sweden; 8 The Norwegian Chiropractors’ Research Foundation «Et Liv I Bevegelse», Lilleakerveien 31, 0283 Oslo, Norway; 9 Department of Health and Functioning, Faculty of Health and Social Sciences, Western Norway University of Applied Sciences, Bergen, Norway; 10 Université de Paris-Saclay, Gif-sur-Yvette, France; 11 Advanced Physiotherapist Practitioner, University of Nottingham, A25 Academic Rheumatology, Clinical Sciences Building, City Hospital, Nottingham, UK; 12 Synthesis, Economic Evaluation and Decision Science (SEEDS) Group, Health Technology Assessment Unit | Applied Health Research Hub, University of Central Lancashire, Preston, UK; 13 Methodological Innovation, Development, Adaptation and Support (MIDAS) Group, NIHR Applied Research Collaboration North West Coast (ARC NWC), Liverpool, UK; 14 Center for Muscle and Joint Health, Department of Sports Science and Clinical Biomechanics, University of Southern Denmark, Odense, Denmark; 15 Medical Research Unit, Spine Centre of Southern Denmark, Lillebaelt Hospital, University Hospital of Southern Denmark, Middelfart, Denmark; 16 School of Health Sciences, Medical School, Queen’s Medical Centre, University of Nottingham, Room B228a, Nottingham, UK; 17 Division of Physiotherapy and Rehabilitation Sciences, University of Nottingham, Nottingham, England; 18 Department of Health Professions, Faculty of Health and Education, Brooks Building, Manchester Metropolitan University, Manchester, UK; 19 University College of Osteopathy, 275 Borough High Street, London, UK; 20 Health Sciences, Doornfontein Campus, University of Johannesburg, Johannesburg, South Africa

**Keywords:** Spinal manipulation, Chiropractic, Osteopathy, Physiotherapy, Systematic review, Mechanism

## Abstract

**Background:**

Spinal manipulation (SM) has been claimed to change anatomy, either in structure or position, and that these changes may be the cause of clinical improvements. The aim of this systematic review was to evaluate and synthesise the peer-reviewed literature on the current evidence of anatomical changes in response to SM.

**Methods:**

The review was registered with PROSPERO (CRD42022304971) and reporting was guided by the standards of the PRISMA Statement. We searched Medline, Embase, CINAHL, AMED, Cochrane Library all databases, PEDro, and the Index to Chiropractic Literature from inception to 11 March 2022 and updated on 06 June 2023. Search terms included manipulation, adjustment, chiropractic, osteopathy, spine and spine-related structures. We included primary research studies that compared outcomes with and without SM regardless of study design. Manipulation was defined as high-velocity, low-amplitude thrust delivered by hand to the spine or directly related joints. Included studies objectively measured a potential change in an anatomical structure or in position. We developed a novel list of methodological quality items in addition to a short, customized list of risk of bias (RoB) items. We used quality and RoB items together to determine whether an article was credible or not credible. We sought differences in outcomes between SM and control groups for randomised controlled trials and crossover studies, and between pre- and post-SM outcomes for other study designs. We reported, in narrative form, whether there was a change or not.

**Results:**

The search retrieved 19,572 articles and 20 of those were included for review. Study topics included vertebral position (n = 3) facet joint space (n = 5), spinal stiffness (n = 3), resting muscle thickness (n = 6), intervertebral disc pressure (n = 1), myofascial hysteresis (n = 1), and further damage to already damaged arteries (n = 1). Eight articles were considered credible. The credible articles indicated that lumbar facet joint space increased and spinal stiffness decreased but that the resting muscle thickness did not change.

**Conclusion:**

We found few studies on this topic. However, there are two promising areas for future study: facet joint space and spinal stiffness. A research strategy should be developed with funding for high quality research centres.

**Supplementary Information:**

The online version contains supplementary material available at 10.1186/s12998-024-00549-w.

## Background

Spinal manipulation (SM) is an intervention that is commonly sought by people with back and neck pain. Manual therapists, chiropractors and osteopaths, in particular, commonly utilise SM as a therapeutic intervention [1–3]. SM is associated with improved clinical outcomes for certain musculoskeletal disorders [4–7]. As a result, SM is recommended in several treatment guidelines and reviews [8–11]. However, the underlying mechanism(s) of action need to be understood to determine appropriate indications for the application of SM as well as to maximize its therapeutic efficacy. That is, it is important to determine what is inside the “black box” of mechanism(s) of action of SM [12].

There are many theories and assertions on this topic [13], but there is no general consensus on the mechanism(s) of action of SM. It has been claimed that SM can change anatomy, such as repositioning vertebrae [14] or altering the thickness of muscles at rest [15]. It is proposed that these changes may be long-lasting [16]. Other claims include physiological changes, ranging from liberating Innate Intelligence [17], to modification of muscle strength [18] or reducing inflammation [19]. Historically, the nervous system has had a particular interest among chiropractors and osteopaths, as SM has been thought to affect spinal nerves [20, 21], the autonomic nervous system [22], and even the brain [23].

These anatomical and/or physiological changes are then purported to explain any associated clinical improvements, such as increased function, reduced pain, relief from specific diseases, and better health in general [24]. If any of these proposed mechanisms can be supported by evidence, manual therapists will be able to offer to patients a coherent rationale for applying SM.

Any mechanism of manipulation is comprised of two aspects. First, the manipulation must have an effect in the body lasting beyond the application of SM, and this effect must lead to a change in clinical outcome. Both aspects must be investigated in turn to determine potential mechanisms of SM. Although it is possible that there is a cumulative effect from SM or that a minimum dosage is needed to create an effect, this has not been well documented. Therefore, to proceed in a stepwise fashion, it seems reasonable to first determine what the immediate effects may be of one single SM.

There is evidence on what happens within the spine, as a response to various forces applied *during* a high-velocity low-amplitude (HVLA) SM [25–28], such as the distribution of forces within tissues receiving the manipulation [29], and the amount and direction of displacement of vertebrae during SM [30], but a clear picture of what happens directly afterwards appears to be lacking.

Although there have been systematic reviews on some physiological effects of SM [31–33], to our knowledge, there are no systematic reviews that have attempted to synthesise evidence of the underlying anatomical mechanisms of SM. Therefore, we assessed the state of evidence of a measurable change anatomical structures that occurs following the application of SM.

The overall aim of this systematic review was to evaluate and synthesise the peer-reviewed literature on the immediate changes in or to anatomical structures in response to SM.

Our research objectives were as follows:

1. Identify, evaluate the quality of, and narratively synthesise the evidence that has been published in peer-reviewed research literature regarding immediate anatomical change after a spinal manipulation.
2. Identify gaps in understanding the anatomical effects of spinal manipulation and provide recommendations for future research.

## Methods

### Advisory board

A research project advisory board was convened for support and guidance, consisting of a chair (chiropractor KJY), an information specialist (CH), 2 chiropractors (CLY, IA), a physiotherapist (RK), an osteopath (SV), a medical doctor/chiropractor (PS), and an anatomist/orthopaedic surgeon (OG). Several had experience with systematic reviews.

### Team and roles

In all, 14 people (6 chiropractors, 6 physiotherapists, and 2 osteopaths) were recruited to perform the screening of articles. Several team members practice clinically. One reviewer dropped out before screening was completed and was replaced by KJY. Another reviewer dropped out after the screening process and was replaced by LG. One researcher with a chiropractic background, experienced in systematic reviews (CLY), acted as referee and supervisor only. The screening of articles was divided between 7 teams of 2 people each.

### Protocol registration and reporting

The review was registered with PROSPERO (CRD42022304971) and the reporting was guided by the standards of the Preferred Reporting Items for Systematic Review and Meta-Analysis (PRISMA) Statement [34].

### Search strategy

We performed a broad search to capture as many relevant articles as possible and developed our search strategies with an experienced information specialist (CH). The search strategy included relevant subject headings and search terms relating to manipulation and the spine and was adapted for use in each database. We had no resources for a translation service, so we limited the search to the English language. We searched the following databases: Medline (Ovid), Embase (Ovid), CINAHL (EBSCOhost), AMED (EBSCOhost), Cochrane Library all databases (via Wiley), PEDro (https://pedro.org.au/), and the Index to Chiropractic Literature (https://www.chiroindex.org/). All databases were searched from inception to 11 March 2022; the searches were updated on 06 June 2023. The full search strategy can be found in Additional File 1.

In cases where full-text articles were not available through library services, we emailed the first author, if an email address was published in the article. If there was no email address listed, or if we received no response to an email query, we searched for the first author on ResearchGate and, if found, we sent a full-text article request. If there was no response or the author could not provide us with an article, it was excluded.

### Terminology

We used the term “outcome variable” to represent what the researchers measured in each study, referring to the mechanism of manipulation being studied. For instance, if SM is hypothesised to improve a clinical outcome by restoring the position of a vertebra, the mechanism by which SM achieves this end outcome is by changing the position of a vertebra. The outcome variable measured in the experiment would, therefore, be the difference in vertebral position from pre- to post-manipulation. In relation to research findings, we used the term “positive” not as a value judgment, but rather as shorthand to denote when a post-SM change in measurement was reported, and the term “negative”, when no such change was reported.

### Eligibility criteria

We included only peer-reviewed articles if they fulfilled certain criteria:

1. We included primary research studies that compared non-treated with treated anatomical structures, regardless of the study design. The articles had to define SM as an HVLA thrust delivered by hand to the spine or directly related joints (i.e., including the sacroiliac or costo-vertebral joints). The measurement of effect must have occurred after a single manipulation session, that is, not after a course of care. If articles did not state a specific time interval between SM and post-SM measurement, but their research designs, or the way the text was written gave the distinct impression that there was little delay between the manipulation and the post-SM measurement, they were included.
2. The SM could not have been combined with any other therapeutic interventions.
3. Studies must have objectively measured a potential change in anatomical structure (the physical attributes of one or more structures in the human body) or a change in position (the relationship of two or more structures to each other). Studies measuring range of motion were considered subjective and were not included, since participants or assessors could consciously or subconsciously influence the position during the measurement.
4. Anatomical change was considered to be distinct from change in physiological state. Therefore, we included articles that reported on resting muscle thickness, as opposed to contracted muscle thickness, because we considered muscle contraction to be a matter better considered under physiological effects of SM. It is possible that a change in resting muscle thickness may be due to a physiological process such as contraction/relaxation. However, there may also be a purely physical mechanism such as stretching. Therefore, we included it.
5. Animal studies were included, because objectively measured anatomical effects of SM are not subject to contextual effects as clinical outcomes may sometimes be.

If studies measured more than one outcome, only the relevant outcome(s) were considered for this review. For the full list of exclusion criteria, please see Additional File 2.

### Article selection

All articles retrieved through the literature searches were exported into EndNote X9.3.1 (Clarivate, Philadelphia, 2013). After duplicates were removed, the remaining articles were imported into the web-based Rayyan systematic review management application [35] for reference management and tracking of the screening process. The total number of articles was divided into 7 separate reviews on Rayyan, each given to a pair of reviewers to independently screen titles/abstracts. Full-text versions of the potentially included articles were obtained for screening by the same teams. Detailed written instructions were distributed to the reviewers prior to the screening processes and meetings were held with each pair prior to title/abstract screening to facilitate congruence in approach. In cases of disagreement between reviewers during phase one (title/abstract) or phase two (full text) screening, a third independent reviewer (KJY or LG) was consulted to achieve consensus. Finally, KJY and CB conducted a backward search, manually searching the reference lists from all articles included at the full-text screening stage for any missing articles.

### Data extraction

The articles were grouped by topic, and reviewers self-assigned, as much as possible, to 1 or 2 topics, with 2 reviewers independently reviewing each topic. The reviewers were LN, DE, KJY (2 topics), RK, CB, CM, SH, VG, LG, EL GG, KCL, and CN. Each topic pair was overseen by a third reviewer, either KJY, LG or DE, who had knowledge of the topic and was designated as “leader” of the group. Calibration sessions were held by KJY with each team prior to data extraction to help ensure congruency of approach. Study descriptions, methodological quality, and risk of bias (RoB) data were extracted. The 7 pairs of reviewers independently extracted data, with conflicts resolved by discussion between them or with the leader. Findings for each team were reviewed by the leaders of each team. KJY, CLY, and DE reviewed all findings.

### Extracted data

#### Article descriptions

Descriptive information about each article was entered into a table. This included first author/year of publication, mechanism of spinal manipulation investigated, study design, study setting, study cohort, sample sizes of intervention and control groups, control group description, spinal region studied, outcome variable used, instrument used for measurement, and the time interval between SM and measurement.

#### Quality assessment and risk of bias

Because the articles we included had used objective measurements of anatomical/positional outcome variables, there were potential areas of technical error introduced during the experiments. For this reason, and informed by a previous publication [22], we considered the techniques used to study the various outcome variables, and developed a novel list of methodological quality items. These items related to technical aspects of the experiments and transparency in methods.

A standard RoB reporting tool was not applicable due to the heterogeneity of study designs. Further, RoB tools are suitable mainly for clinical studies, in which the influence of the study participant is important to account for. However, in the investigations included in our review, study participant influence would be absent, as they would not likely be able to influence technical readings relating to anatomical structures, either consciously or subconsciously. Therefore, study participants did not need to be blinded to treatment or control group allocation. They also did not need to be naïve to the treatment. Thus, we included RoB items only relating to the blinding of assessors and statisticians. We selected only RoB items that we considered appropriate for the relevant study designs, i.e., depending on if they had a control group or not. We also included random allocation when two interventions were compared because it was important that inherent differences between groups was eliminated. We then considered those quality and RoB items together to determine, whether an article was credible or not credible, in a process described below.

#### Results

Results of each study were extracted, after the quality and RoB items were determined, to avoid reviewer bias of the quality/RoB assessment. The results of each of the studies were extracted from the articles by 3 members of the team. CM reviewed half the articles and SV reviewed the other half, each working in conjunction with KJY, who reviewed all articles. Conflicts and queries were resolved by discussion or consultation with CLY. Results were entered into separate tables for each outcome variable, including the ultimate finding on whether the anatomical structure was affected by the manipulation or not. All tables were consolidated and edited for readability, and each team reviewed and approved their consolidated tables.

### Data synthesis

#### Assessment and weighting of quality and risk of bias

Through consensus discussion, we defined the quality and RoB items by the consequences we assumed that they would have on the credibility of the data. For this purpose, we developed a dichotomous system of weighting. Items were determined to be either “critical” or “important”. “Critical” items were essential to the credibility of the results, whereas “important” items were those that were considered good practice but were not essential, in and of themselves, to a judgement on whether results could be considered credible.

#### Quality items

We considered 3 quality items to be “critical”. First was “evaluation tool(s) appropriate to measure outcome variable(s)”. The second critical item was “reported the reliability of outcome variable(s)”. The final critical quality item was “measurement tool calibrated” (if appropriate). The remaining quality items on our checklist were assessed as important, but not critical.

#### Risk of bias items

For non-RCT studies, we included only one RoB item, “assessor blinding to pre-post manipulation status”, and we considered it to be critical. For RCTs and crossover study designs, 2 RoB items were considered critical. First was “random allocation of participants”. The second was “assessor blinding to intervention group”. The remaining RoB item was “statistician blind to intervention/control group”, which is not commonly reported in articles. For this reason, we decided to classify it as important rather than critical.

#### Assessment of the certainty of evidence

We used the quality and RoB tables to establish “credibility” for each article and outcome variable. If a quality or RoB item was appropriately reported, it was left as white in the table. However, items that had not been reported or were poorly reported, were marked as yellow for important items and red for critical items.

We then made an overall assessment of credibility for each article, based on both the quality and RoB, after which each article was defined as “credible” or “not credible”. Articles were defined as credible if they had 0 red *and* 0–2 yellow items. Articles were defined as not credible if they had 1 or more red items *or* 3 or more yellow. Please see Table 1 for the key to interpreting the quality and RoB items as well as explanations of each.

**Table 1:**
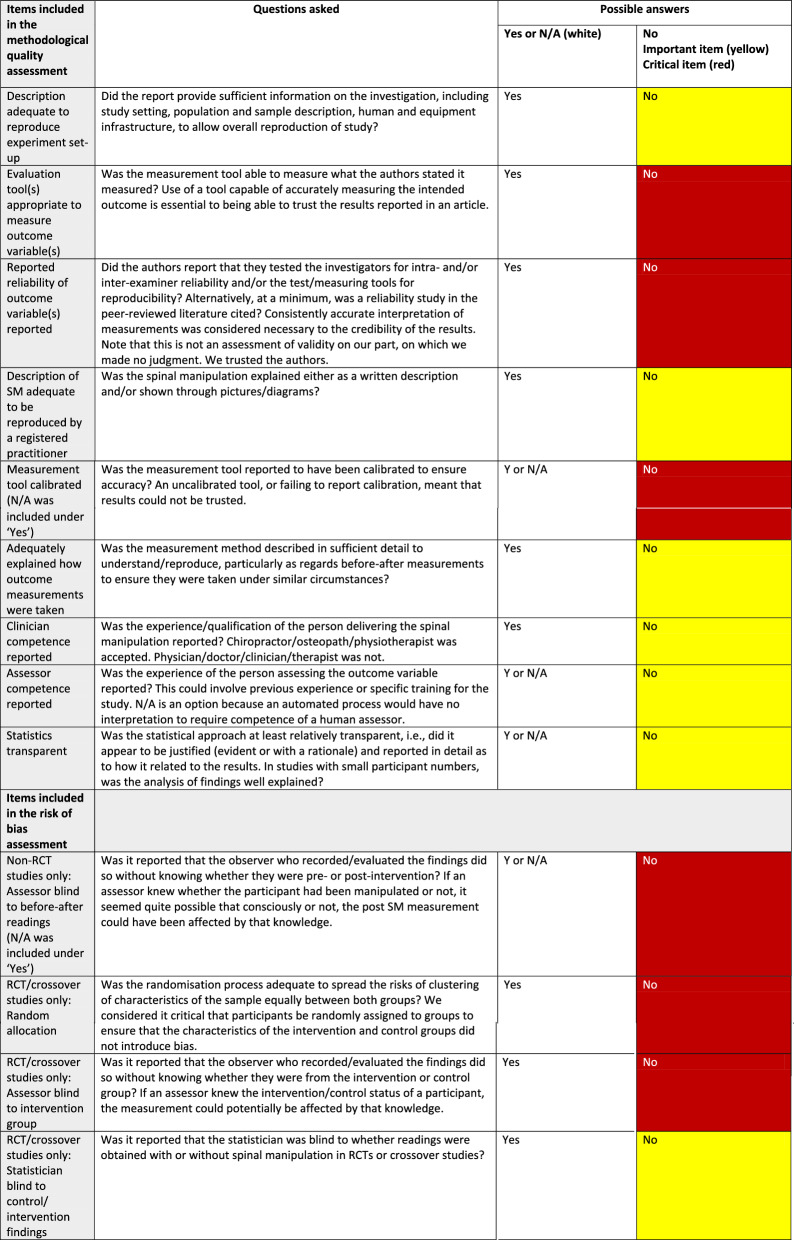
Key to interpreting the quality/risk of bias tables in a systematic review of anatomical mechanisms of spinal manipulation

N/A: not applicable

RCT: randomised controlled trial

#### Reporting of results

It was not suitable to pool the results for meta-analysis due to heterogeneity in outcome variables, study design, and participant characteristics. It was also not relevant, because our research question did not require a measurement (e.g., amount of facet joint space increase or cross-sectional area increase in muscle size) but rather just the presence or absence of change post-SM. These results were reported both in detail and summarized in tables as “positive” (i.e., there was a change post-SM) or “negative” (i.e., there was no change).

To excerpt maximum information from this research area, we reported results from all studies, but separately for the “credible” and “not credible” articles. We also included a summary of methodological issues in the Discussion to aid future researchers to improve this research area. Our rules on reporting data were as follows:

(i) We reported differences between SM and control groups for RCTs and crossover studies and between pre- and post-SM groups for other study designs. When there were several results reported in one article, we used the “best” estimates (i.e., best case scenario of a “positive” outcome or difference pre-post SM). For example, one article reported a positive result when the participant was re-measured while remaining in side-posture position, but there was a “negative” result when the participant was returned to neutral (supine) position for post-SM measurement. In this case, we reported the positive result.
(ii) If there were no statistically significant differences or statistical significance tests were not reported, we provided, again, the “best” estimate (i.e., selecting the best-case scenario).
(iii) If no estimates (direct measurements) were reported, but rather only significance values, then we reported those.

### Writing and editing the manuscript

To keep the workload achievable and to improve attention to detail in manuscript development, we used an iterative process. Each section of the paper (introduction, methods, results, and discussion) and all tables and figures were disseminated to the research team for comments at different stages. In cases of disagreement, an appropriate member or members of the advisory board were contacted, and discussions were held amongst team members until consensus was reached.

## Results

### General

**Fig. 1:**
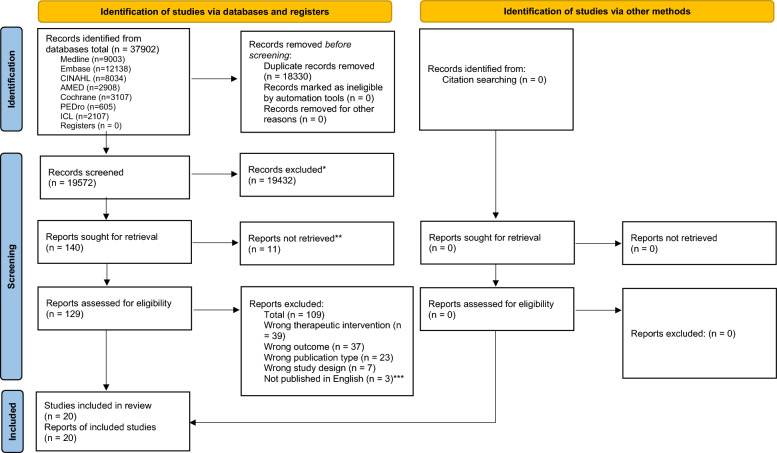
PRISMA 2020 flow diagram for new systematic reviews which included searches of databases, registers and other sources. * Records were excluded by humans; no automation tools were used. ** Eleven articles were not available for full-text retrieval after having unsuccessfully attempted to contact the first author of each in two ways, as detailed in the Methods section. *** Three articles were retrieved which had their titles/abstracts in English but the manuscripts in a foreign language and thus were excluded at the full-text screening stage

Of the original 19,572 articles (37,902 including duplicates), 20 articles that reported on 20 studies were ultimately included in this review (Fig. 1).

### Study descriptions

As shown in Table 2, the reviewed articles included the following outcome variables: vertebral position (n = 3) [14, 36, 37], facet joint space (n = 5) [38–42], spinal stiffness (n = 3) [43–45], resting muscle thickness (n = 6) [15, 46–50], intervertebral disc pressure (n = 1) [51], myofascial hysteresis (n = 1) [52], and further damage to damaged arteries (n = 1) [53].

**Table 2:** Descriptions of 20 studies included in a systematic review of anatomical mechanisms of spinal manipulation

First author year of publication	Mechanism(s) of spinal manipulation investigated	Study design	Study setting	Study cohort	Sample size: – N cases – N controls (or control interventions)	Control group description	Spinal region(s)	Outcome variable(s)	Instrument(s) used for measurement	Time interval between spinal manipulation and measurement
Jirout 1972	Change in vertebral position	Uncontrolled intervention	–	Humans with palpatory finding of a “blocade” on lateral flexion of neck	250: − 250 − 0	N/A	Cervical	Vertebral segment position change	Plain radiography	–
Cramer 2000	Increase in facet joint space	RCT	Chiropractic school, USA	Healthy adults, 22–29 yrs	16: − 8 (2 groups of 4) − 8 (2 groups of 4)	(i) Neutral position (ii) Side posture position	Lumbar	Facet joint space change	MRI	“Immediately”
Cramer 2002	Increase in facet joint space	RCT	Chiropractic school, USA	Healthy adults, 22–30 yrs	64: − 32 (2 groups of 16) − 32 (2 groups of 16)	(i) Neutral position (ii) Side posture position	Lumbar	Facet joint space change	MRI	“Immediately”
Cascioli 2003	(i) Increase in facet joint space (ii) Formation of intra-articular gas bubbles	Uncontrolled intervention	Medical teaching hospital, South Africa	Chiropractic students, 23–24 yrs, without cervical complaints	2: − 2 − 0	N/A	Cervical	(i) Facet joint space change (ii) Intra-articular gas bubble formation	Plain radiography	“Immediately”
Lisi 2006	Change in intervertebral disc pressure	Feasibility	-	Healthy adults, 41–42 yrs	1: − 1 − 0	N/A	Lumbar	Intervertebral disc pressure	Pressure sensor probe	Immediately and for 15 s after spinal manipulation
Brenner 2007	Change in multifidous muscle thickness	Case report	Army facility, USA	Male, 33 yrs, chronic LBP	1: − 1 − 0	N/A	Lumbo-pelvic	Thickness of multifidus at rest	Diagnostic ultrasound	“Immediately”
Haussler 2007	Change in spinal stiffness	Crossover	Equine research park at a university, USA	Healthy adult horses with 2 pins in spinous processes to simulate acute back pain	10: − 10 − 10	7-day washout before crossover; Control was no intervent	Thoracic Lumbar	Spinal stiffness	Cable extensometer Pressure sensor mat	–
Raney 2007	Change in lateral abdominal muscle thickness	Prospective case series	Army facility, USA	Adults, 18–53 yrs, acute/subacute LBP, SMT responders	9: − 9 − 0	N/A	Lumbo-pelvic	Thickness of lateral abdominal muscles at rest	Diagnostic ultrasound	“Immediately”
Wynd 2008	Further damage to already damaged arteries	Uncontrolled intervention	University lab, Canada	Dogs	10: − 0 − 0	N/A	Cervical	Increase in dimensions of vascular lesion	Intravenous diagnostic ultrasound Fluoroscopy	-
Palmer 2009	Change in vertebral position	Retrospective case series	Private chiropractic clinic, USA	Chiropractic patients, 18–65 yrs, non-migraine headache	35: − 35 − 0	N/A	Cervical	Vertebral segment position change (“atlas laterality”)	Plain radiography	“Immediately”
Cramer 2011	Increase in lumbar facet joint space	Feasibility	Chiropractic school, USA	Healthy adults, 25–27 yrs	5: − 5 − 0	None, but cavitated joints were compared with non-cavitated	Lumbar	Facet joint space change	MRI for gapping; Accelerometer for cavitation	“Immediately”
Fritz 2011	Change in spinal stiffness	Prospective case series	–	Physiotherapy patients, 19–60 yrs, LBP with or without leg symptoms	51: − 50 − 0	N/A	Lumbo-pelvic	Spinal stiffness	Mechanical indentation device	“Immediate”
Konitzer 2011	Change in thickness of transverse and internal oblique abdominal muscles	Prospective case series	Army facility, USA	11F, 8M, 21–46 yrs, chronic LBP who met a clinical prediction rule for lumbar stabilization	19: − 19 − 0	N/A	Lumbo-pelvic	Thickness of transverse and internal oblique abdominal muscles at rest	Diagnostic ultrasound	“Immediately”
Puentedura 2011	Change in thickness of transverse abdominal muscles	Crossover, randomised	University, USA	19F, 16M, 21–34 yrs, asymptomatic university staff and students	35: − 35 − 35	19F, 16M, 21–34 yrs, asymptomatic university staff and students	Lumbar	Thickness of transverse abdominal muscle at rest	Diagnostic ultrasound	“Immediately”
Cramer 2012	Increase in facet joint space	RCT	Chiropractic school, USA	Healthy adults, 18–30 yrs	40: − 30 − 10	No SMT; Also cavitated joints were compared with non-cavitated	Lumbar	Facet joint space change	MRI for measuring facet joint space; accelerometer for cavitation	“Immediately”
Barnes 2013	Change in myofascial hysteresis	RCT	Private osteopathic college lab, USA	NR	80: − 40 − 40	NR	Cervical	Change in tissue texture characteristics	Durometer	“Approx 10 min”
Wong 2015	Change in spinal stiffness	Non-randomised controlled study	–	18–60 yrs, LBP, SMT responders	107: − 32 (15 previously identified SMT responders, 17 non-responders) − 75	(i) 59 asympto-matic (to session 1); 57 for sessions 2 and 3 who did not receive SMT − 16 w/LBP that did not receive SMT	Lumbo-pelvic	Spinal stiffness	Mechanical indentation device	“Immediate”
Haavik 2016	Change in pelvic floor muscle thickness	Crossover, unrando-mised	Chiropractic school, New Zealand	Healthy pregnant, 18–38 yrs	26: (i) 11 pregnant (ii) 15 non-pregnant (i) 15 non-pregnant (ii) 11 pregnant	Non-pregnant chiro-practic students, 19–32 yrs	Unspeci-fied spine and/or pelvis	Thickness of levator hiatus muscles at rest	Transperineal diagnostic ultrasound	(i) Same day for pregnant participants (ii) Not reported for nonpregnant
Flaum 2017	Change in vertebral position	RCT	Medical school, USA	Asymptomatic medical students, 22–40 yrs	51: − 25 − 26	Asympto-matic medical students, 22–40 yrs	Cervical	Tissue depth to articular pillar compared bilaterally (proxy for vertebral rotation)	Diagnostic ultrasound	“Immediately”
Fosberg 2019	Change in transverse abdominal muscle thickness	RCT	University and private clinic, USA	18–70 yrs, LBP, SMT responders	67: − 33 − 34	18–70 yrs, LBP, SMT responders	Lumbar	Thickness of transverse abdominal muscles at rest	Diagnostic ultrasound	“Immediately”

LBP: Low back pain, MRI: Magnetic resonance imaging, N/A: Not applicable, RCT: Randomised controlled trial, SMT: Spinal manipulative therapy

Study designs included both controlled (n = 10) and uncontrolled (n = 10) studies. The number of study participants ranged from 1 to 250. The most commonly studied participants were healthy adults (n = 9). Two studies included animals.

Most studies (n = 12) took place in the United States of America, with a private chiropractic or osteopathic school as the most common setting (n = 6), while 3 studies explicitly stated that they used a lab setting. SM was most commonly performed on the lumbar spine or lumbopelvic area (n = 12).

#### Methodological quality

As can be seen in Tables 3, 4, 5, 6, and 7, issues relating to the methodological quality varied. Regarding the two critical quality items, the first, “using an appropriate evaluation tool” was consistently lacking in the 3 articles on vertebral position, and the second, “reporting of the reliability of an outcome variable” was not present in 6 of the 11 articles.

**Table 3:**
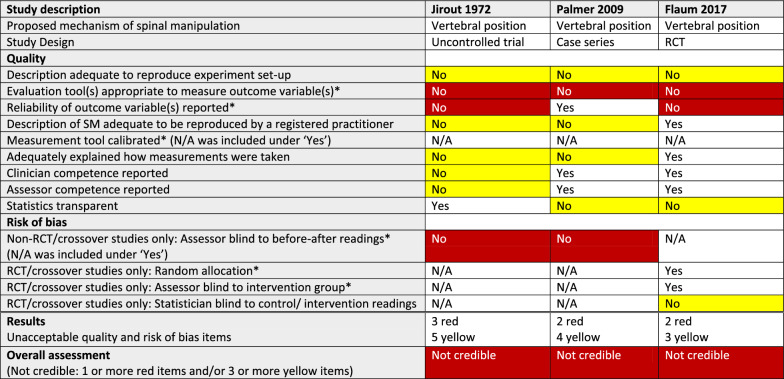
Quality, risk of bias, and credibility assessments for 3 articles on vertebral position in a systematic review of anatomical mechanisms of spinal manipulation

^*^Critical item (no asterisk: important item)

White: requirement fulfilled (Yes) or not applicable (N/A)

Red: Critical quality or risk of bias item not fulfilled (No)/Not credible article

Yellow: Important quality or risk of bias item not fulfilled (No)

**Table 4:**
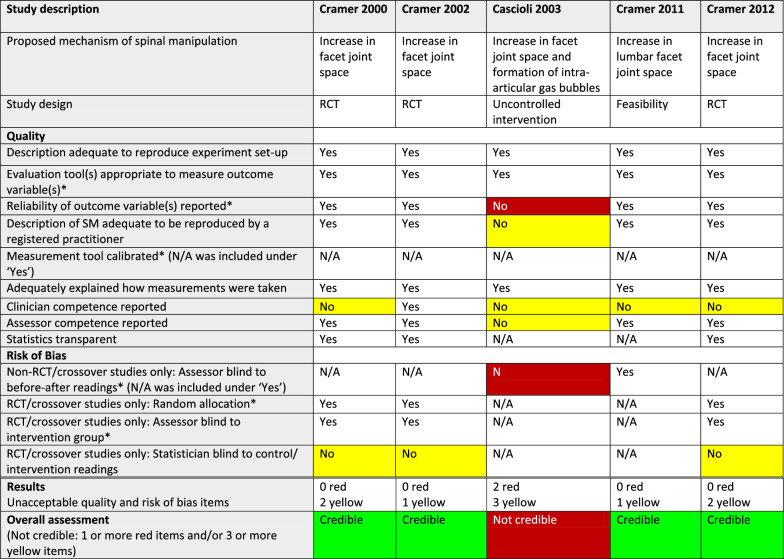
Quality, risk of bias, and credibility assessments for 5 articles on facet joint space in a systematic review of anatomical mechanisms of spinal manipulation

^*^Critical item (no asterisk: important item)

White: requirement fulfilled (Yes) or not applicable (N/A)

Red: Critical quality or risk of bias item not fulfilled (No)/Not credible article

Yellow: Important quality or risk of bias item not fulfilled (No)

Green: Credible article

**Table 5:**
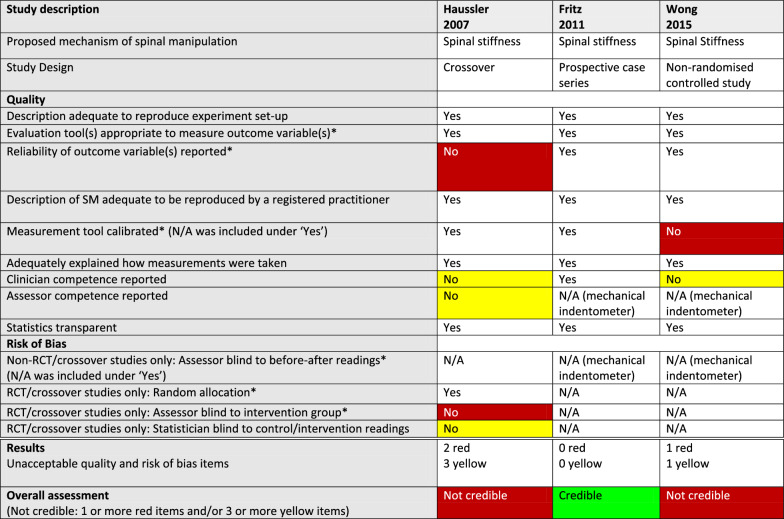
Quality, risk of bias, and credibility assessment for 3 articles on spinal stiffness in a systematic review of anatomical mechanisms of spinal manipulation

^*^Critical item (no asterisk: important item)

White: requirement fulfilled (Yes) or not applicable (N/A)

Red: Critical quality or risk of bias item not fulfilled (No)/Not credible article

Yellow: Important quality or risk of bias item not fulfilled (No)

Green: Credible article

**Table 6:**
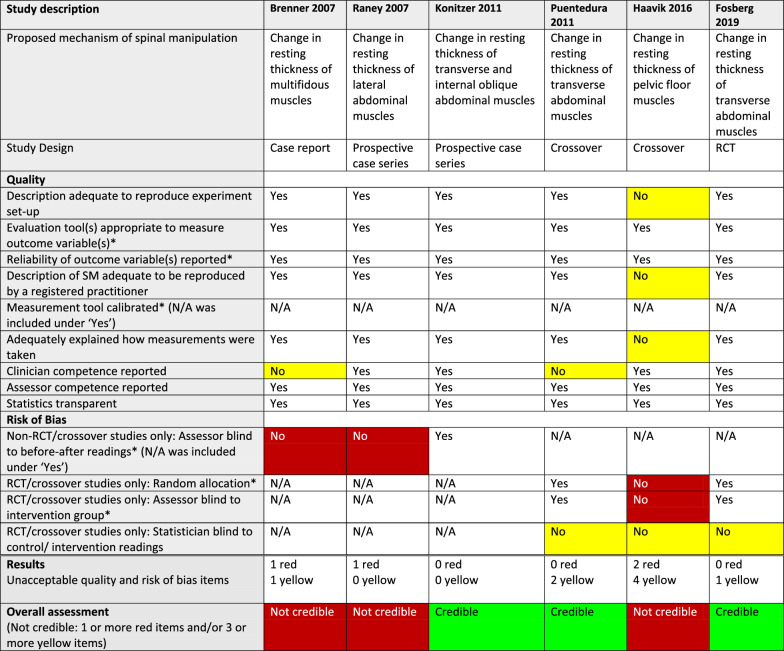
Quality, risk of bias, and credibility assessment for 6 articles on muscle thickness in a systematic review of anatomical mechanisms of spinal manipulation

^*^Critical item (no asterisk: important item)

White: requirement fulfilled (Yes) or not applicable (N/A)

Red: Critical quality or risk of bias item not fulfilled (No)/Not credible article

Yellow: Important quality or risk of bias item not fulfilled (No)

Green: Credible article

**Table 7:**
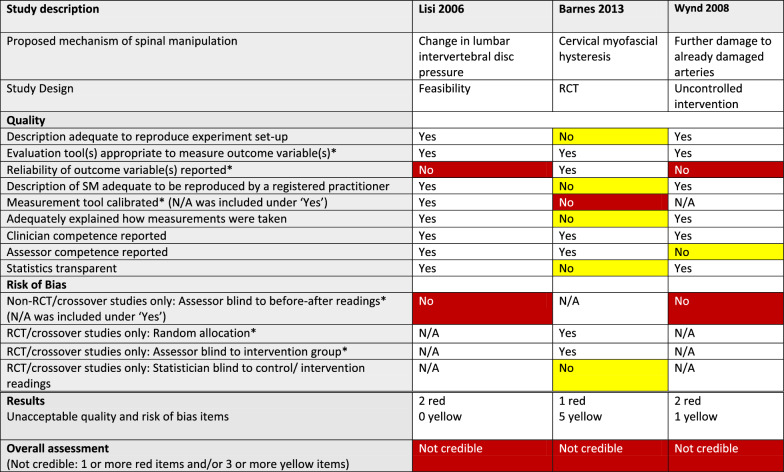
Quality, risk of bias, and credibility assessment for 1 article on intervertebral disc pressure, 1 article on cervical myofascial hysteresis, and 1 article on further damage to already damaged arteries in a systematic review of anatomical mechanisms of spinal manipulation

^*^Critical item (no asterisk: important item)

White: requirement fulfilled (Yes) or not applicable (N/A)

Red: Critical quality or risk of bias item not fulfilled (No)/Not credible article

Yellow: Important quality or risk of bias item not fulfilled (No)

#### Risk of bias

We did not find that critical RoB was a problem in studies using an RCT or crossover design (Tables 3, 4, 5, 6, and 7), as 7 of 9 such articles had no critical deficiencies in this domain. However, in other study designs, 7 of 11 articles did not report that outcome measurements had been taken by assessors who were blinded to the previous assessment.

#### Credibility

The RoB/quality tables (Tables 3, 4, 5, 6 and 7) show that 8 of the 20 studies were considered credible, whereas the other 12 were considered not credible. Notably, two of the studies, reported by Konitzer [46] and Fritz [43], met all our quality and RoB criteria.

The numbers of articles reporting on credible studies were as follows: facet joint space (n = 4/5), spinal stiffness (n = 1/3), and resting muscle thickness (n = 3/6). Therefore, 1 of the 5 studies on facet joint space was not credible; 2 of the 3 studies on spinal stiffness were not credible and 3 of the 6 studies on resting muscle thickness were not credible. In addition, *all* the articles reporting on studies on vertebral position (n = 3), intervertebral disc (IVD) pressure (n = 1), further damage to damaged arteries (n = 1), and myofascial hysteresis (n = 1) were found to be not credible.

#### Results of credible studies by outcome variable

Results from the 8 credible studies are shown below, reported by outcome variable.

##### Facet joint space (n = 4/5)

Four of 5 studies on changes to facet joint space were considered credible. They all reported an increase in lumbar spine facet joint space post-side-posture manipulation for the “up” side facet joints, but only if the participant was re-scanned using magnetic resonance imaging (MRI) while maintaining side posture position. When returned to neutral position the increased joint space disappeared.

##### Spinal stiffness (n = 1/3)

Only 1 of the 3 studies on spinal stiffness was found credible and reported immediate reduced spinal stiffness post-SM.

##### Resting muscle thickness (n = 3/6)

Three of six studies on changes to resting muscle thickness were considered credible. These studies, using diagnostic ultrasound, reported no statistically significant differences in either the transverse or internal oblique abdominal muscles post-manipulation.

#### Results of not credible studies by outcome variable

Below are shown the results from the 12 not credible articles, reported by outcome variable.

##### Vertebral position (n = 3/3)

The results of these 3 not credible studies were conflicting; 2 articles reported post-SM changes in vertebral position, using plain radiography, whereas one reported no change post-SM using ultrasound.

##### Facet joint space (n = 1/5)

The 1 not credible study had only 2 participants measured for the relevant outcome variable as part of a larger study, in which all other participants also received traction before and after SM, and so were not considered controls. No change in facet joint space and no presence of pneumarthrosis (discrete bubble of intra-articular gas) was reported post-SM using computed tomography (CT).

##### Spinal stiffness (n = 2/3)

The 2 not credible studies found a reduction in spinal stiffness post-SM. One study used a mechanical indentometer; the other studied horses using a cable extensometer with a pressure sensor mat.

##### Resting muscle thickness (n = 3/6)

Results in the 3 not credible studies were conflicting. One reported a pre- post-SM difference in resting transverse abdominal muscle thickness. One reported a difference in the resting thickness of pelvic floor muscles in pregnant women but not in non-pregnant women. The third reported no difference in thickness for multifidus muscles. All studies made measurements using ultrasound.

##### Intervertebral disc pressure (n = 1/1)

There was 1 not credible study which found increased disc pressure post-SM for at least 15 s using a pressure probe inserted into the disc.

##### Myofascial hysteresis (n = 1/1)

The 1 not credible RCT used a durometer to measure hysteresis with mixed results that we found difficult to interpret.

##### Further damage to already damaged arteries (n = 1/1)

The 1 not credible study on this topic was an uncontrolled intervention. The article reported no further damage post-SM to vertebral arteries of dogs that were damaged prior to manipulation with an angioplasty cutting balloon. This study was stopped early for ethical reasons once no effect was detected.

Please see Table 8 for the results and credibility assessments of all studies.

**Table 8:**
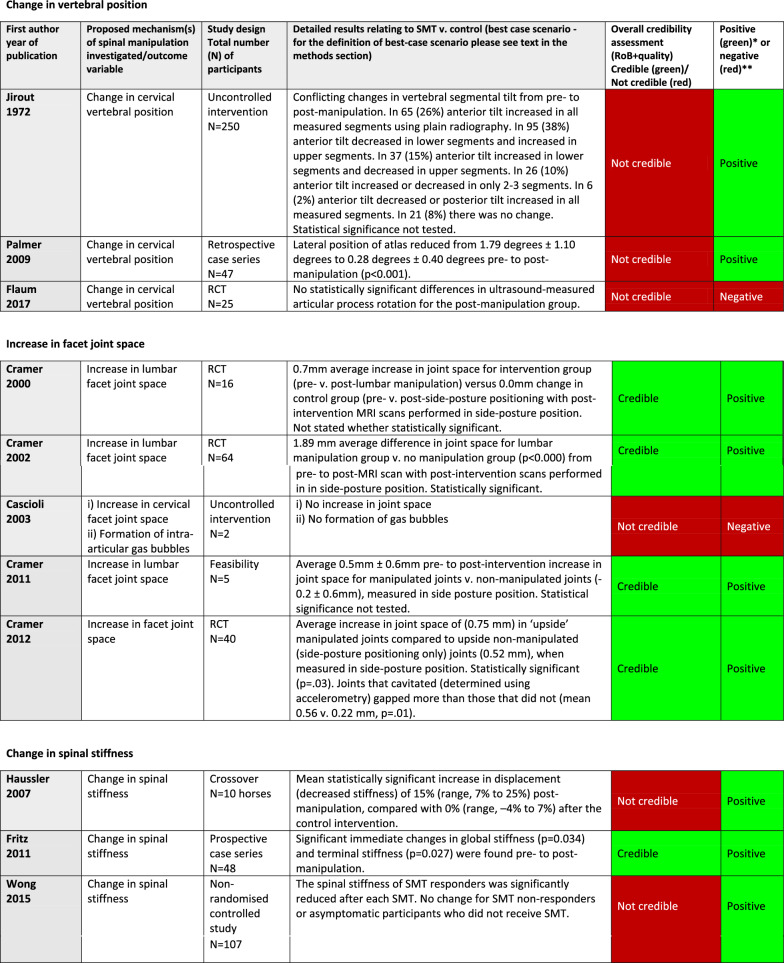

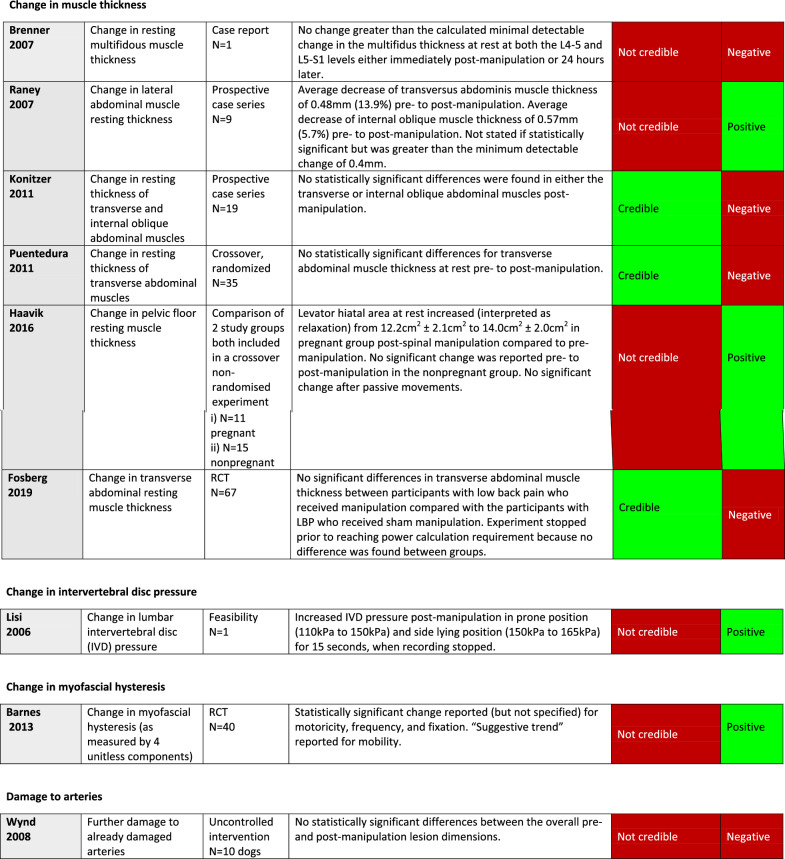
Results reported in 20 articles included in systematic review of anatomical mechanisms of spinal manipulation. Table shows results grouped by proposed mechanism of spinal manipulation (column 2), with detailed results (column 4), overall credibility (column 5), and whether results were positive or negative (column 6)

^*^Positive result: spinal manipulation led to a change

^**^Negative result: spinal manipulation did not lead to a change

#### Post-hoc analysis

Cross-referencing credibility with positive (change after SM) or negative (no change after SM) results of SM resulted in a mixed picture. Thus, there was no relationship between the credibility of studies with positive or negative results of these studies. For a visual summary of the findings by outcome variable, credibility, and whether an article reported positive or negative results, see Table 9.

**Table 9:**
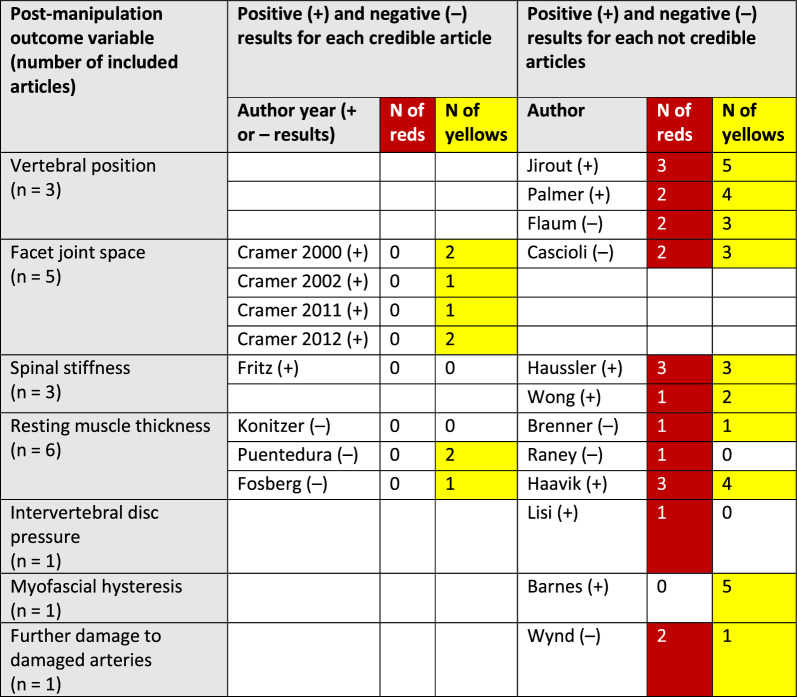
Synthesis of findings by outcome variable and author in a systematic review of 20 articles of anatomical mechanisms of spinal manipulation

Positive (+): article reported a change after spinal manipulation

Negative (−): article reported no change after spinal manipulation

Red: critical quality or risk of bias item(s)

Yellow: important quality or risk of bias item(s)

## Discussion

### Summary of main findings

This systematic review included 8 articles that we considered to be credible and 12 that we regarded as not credible. They dealt with 7 proposed SM mechanisms of action: change in vertebral position, facet joint space, spinal stiffness, resting muscle thickness, IVD pressure, myofascial hysteresis, and further damage to damaged arteries. We include results from articles that we considered credible as well as ones we considered not credible, in order to report as fully and fairly as possible, any information published to this point on the immediate anatomical/positional changes in response to SM.

The credible articles reported that, post-SM, there were: (i) changes in facet joint space, (ii) changes in spinal stiffness, but (iii) no changes in resting muscle thickness after SM.

A comparison between the results of the credible and not credible articles revealed that the latter: (i) disagreed with the credible articles on facet joint space/pneumarthrosis (discrete bubble of intra-articular gas), (ii) agreed on spinal stiffness, but (iii) had conflicting results for resting muscle thickness.

In addition, the not credible articles reported (i) conflicting results between them on vertebral position, (ii) change in IVD pressure and (iii) change in myofascial hysteresis, but (iv) no further damage to already damaged arteries after SM.

### Comparisons to the literature

To our knowledge, this is the first systematic review dealing exclusively with the immediate anatomical/positional changes in response to SM. However, a systematic review on spinal mobilisation (i.e., not HVLA manipulation) reported on articles that evaluated mainly clinical outcomes but also on some anatomical mechanisms [33]. Specifically, they included 4 articles that evaluated spinal stiffness, and 3 of the 4 reported reduced spinal stiffness after mobilisation (not HVLA SM). These findings thus aligned with the results in our 3 credible articles that reported reduced spinal stiffness post-SM.

#### Methodological considerations of the present systematic review

##### Literature search

Important positive aspects of our literature search were that an experienced information specialist (CH) ensured that we consulted all relevant databases for this topic and that all databases were interrogated using the different input parameters necessary to retrieve the relevant articles. We also used broad search parameters with no date limit, to capture all relevant articles on the topic. The search was updated to ensure we captured any more recent articles prior to submission of our review. We limited the search to articles in English only. Although, therefore, we may have failed to include every relevant published article, we believe this to be a minor limitation, as most articles dealing with SM are typically published in the English language. Also, we did not follow the PRESS guideline recommendation [54] to perform a review of the search strategy, which might have affected the quality and comprehensiveness of our search.

##### Inclusion/exclusion criteria

To ensure that SM was the most likely reason for any potential change in an outcome variable, we included only articles on studies that performed pre- and post-SM measurements and reported this for a single SM session. If some effects of SM require multiple sessions to manifest, we would have missed out on such information. We also excluded articles that used SM in combination with any other therapeutic intervention. Therefore, if SM requires facilitation by another intervention for the effects to manifest, we would have missed those changes. There may have been differences among team members in interpretation of inclusion/exclusion criteria which could have led to articles being missed out, although we mitigated this possibility with calibration sessions and written instructions, so this is unlikely.

##### Full text screening

Eleven articles included at the title/abstract screening stage were not available for the full-text screening process. Nine of those were published in chiropractic subluxation-focused journals, thus not available through mainstream library systems, with authors either not found on ResearchGate or not responding to requests for articles. This missingness of data may have affected our conclusions, but our experience is that the “grey” chiropractic journals do not attract high quality articles, so we do not believe that our conclusions were impacted.

##### Quality/risk of bias

It is well known that clinical studies will more easily produce positive findings if the human factor is allowed to play an essential role by voluntarily or subconsciously introducing bias. This is the reason why systematic reviews assess the RoB before drawing conclusions on the validity of results. This phenomenon was clearly shown in a previous systematic review on the “effect” of spinal manipulative therapy (SMT) on non-musculoskeletal conditions [55]. All studies that were considered to have failed in preventing the “human factor” reported positive results, whereas none of the high-quality studies found there to be an “effect” of SMT [55].

We emphasized RoB only in relation to aspects that clearly could be influenced by the beliefs and wishes of the researchers. Experimental/basic science (e.g. laboratory) studies, are susceptible to fewer RoB items. Instead, commercial and university laboratories are often subjected to accreditation procedures [56, 57], which are specific to the requirements of their area of activities and relate more to technicalities than to systematic human errors. Thus, the use of appropriate and calibrated tools that are operated by competent personnel would be paramount in preventing random errors in studies that rely on technical assessments, beyond that of human subjective observations and reactions.

Results in studies that deal with anatomical/positional changes after SM are, thus, unlikely to be influenced by the beliefs and wishes of study participants and clinicians, whereas the beliefs and wishes of the assessor and statistician could have an impact. Failing to use a reliable outcome variable, measuring changes with a non-calibrated machine, or allowing amateurs to conduct the study, could possibly induce bias but more likely result in random errors.

Therefore, in addition to the RoB, we accounted for several technical aspects, defined as “quality”. Quality issues are likely to cause non-systematic errors, as opposed to bias, which may cause systematic errors. Examples of quality issues that must be assured to prevent non-systematic errors are skills of assessment personnel and the calibration of measurement tools. To accurately judge the technical quality of studies, we ensured that each review team had at least one member with expertise in the area.

Nonetheless, the evaluation and weighting of methods was a subjective matter, and other investigators may have judged differently. There may also have been differences among team members in the interpretation of quality and RoB criteria, which could have led to differences in results. However, we mitigated that possibility with multiple calibration sessions and repeated consultations on iterations of the tables among the team members.

##### Results of studies

The results were extracted from each article only after the quality and RoB items were determined, to avoid reviewer bias of the quality/RoB assessment. To prevent biased results interpretation, extraction of the study outcomes and interpretation of findings were performed by other teams than the experts in the area, although the experts were invited to critically review the extracted findings and interpretations.

##### Synthesis

Part of the standard synthesis in systematic reviews is to identify ‘gaps in the literature’. However, these ‘gaps’ may not be areas that have not been studied, just areas that have not been studied *well.* By listing all the studies that have been conducted in this area and detailing all the methodological errors that we identified, we show which areas *can* be studied (or which may be too difficult to study with current technology) and also indicate how they may be studied *better* in the future.

#### Methodological considerations for reviewed articles and potential future studies

##### General comments

In the hope of being helpful to future researchers, we provide some methodological comments regarding the techniques used to study the potential anatomical/positional effects of SM.

Only 8 of the 20 studies were assessed as credible. Consequently, this indicates that technical experimental studies have not been prioritised in environments that were created for such purposes, i.e., taking advantage of relevant equipment and skilled researchers working in dedicated research laboratories. Assessors for these procedures should be highly experienced or adequately trained. In addition, when there is a human element, it is important to establish inter-/intra-rater reliability of the assessors, before undertaking the study. Further, researchers must strive towards the use of measurement tools that are validated, and it is important to remember that some need to be calibrated. The use of frameworks like COSMIN (Consensus-based Standards for the selection of health Measurement Instruments) [58] may be helpful in this process. Also, when measuring positions or spaces, it would be of utmost importance to place study subjects in identical positions before and after the SM, to prevent ‘normal’ aberrations and measurement errors due to distortion.

Items relating to relevant RoB should be observed, in particular, blinding of before-after readings in studies without control groups. Statisticians should be blinded to control/intervention readings. Statistical methods, cut points, etc. should be determined a priori, rather than after any results have been returned.

##### Comments relating to each outcome variable

###### Vertebral position

Two of 3 studies on vertebral position used radiographs to try to capture very small post-SM changes (< 2 degrees of rotation [14] or < 4mm of displacement). However, we note that the use of plain radiography to detect very small changes in vertebral position is debatable, at best, and in our opinion, the use of radiography for these measurements was not appropriate [59]. Instead, we suggest that future investigations could use computed tomography, which offers much greater resolution. The precision and likely error tolerance of the measurement instrument should be stated in future studies.

The third study used ultrasound to measure paraspinal tissue thickness as a proxy for vertebral rotation. However, the pressure on the ultrasound head was not measured. This is important, as increased pressure could compress tissues and distort readings. Nevertheless, this method could hold promise for developing a method of measuring vertebral rotation without the use of ionising radiation (Tables 2 and 3).

###### Facet joint space

All 4 credible articles found an increase in facet joint space post-SM. However, as they were conducted by the same team of researchers, these results should be confirmed by at least 1 independent team.

MRI is likely optimal for viewing facet joint space increase that is retained after the SM event, as bone and capsule detail can be captured.

Theories exist on the potential role of SM on intra-articular meniscoids/discoids, synovial folds, and adhesions within the facet joints [60–65]. We found no relevant articles that investigated any of these anatomical variations/pathologies. We suggest that if someone wanted to study them, they may be visualised using high resolution imaging such as MRI (Tables 2 and 4).

###### Spinal stiffness

The use of the mechanical indentometer in 2 of the 3 studies on spinal stiffness was useful because it removed the human element from the interpretation of the measurements. Calibration of the tool is important in this area (Tables 2 and 5).

###### Resting muscle thickness

Since 3 credible studies all showed absence of changes to resting muscle thickness, we believe that this area no longer warrants further study. We found the study design in one of the not credible articles [50] overly complex and difficult to understand; it seemed to report on 2 crossover studies, with important differences between the intervention and control groups (Tables 2 and 6).

###### Intervertebral disc pressure

The 1 study conducted on IVD pressure showed that it is difficult to study. The use of a pressure-measuring probe to physically penetrate the disc is not attractive to study participants and seems ethically challenging, as it damages the disc tissue to an unknown extent. Perhaps an indirect method of measuring IVD pressure could be developed in the future, for instance using fluid diffusion into or out of the disc, as measured by MRI, as an indicator of pressure. Alternatively, perhaps candidates for IVD replacement could be included as participants (Tables 2 and 7).

###### Further damage to already damaged arteries

In vivo studies of arterial walls are also difficult to perform. In addition, it seems unlikely that SM can cause arterial damage de novo [66–72]. The assumption tested in this article is interesting, if one thinks that it is mainly arteries with pre-existing damage or pathology that are susceptible to further damage by SM. Hence, the authors devised a method to “pre-damage” vertebral arteries in dogs, as a proxy for “naturally occurring” damage or pathology. The types of lesions created were not predictable, for which reason the usefulness of this method is unclear.

The assessment method in this study was complex, using a fluoroscopically guided ultrasound probe as the measurement tool. However, magnetic resonance angiography would offer better resolution, or a micro-video-camera could allow direct visualisation of arterial damage.

Since vertebral artery dissection is so rare and only temporally linked to SM [73, 74], the justification of sacrificing animals to study this should be considered (Tables 2 and 7).

###### Myofascial hysteresis

There were several unclear elements in this article, making it difficult for us to interpret how well the outcome variables in the article related to the concept of hysteresis (Tables 2 and 7).

## Conclusions

### Clinical perspectives

Although this review is primarily valuable to researchers, clinicians should also benefit from our findings. It is a common clinical observation that patients can experience sudden relief immediately after SM. In our experience, when this happens, they may ask: “What exactly happened when you cracked my back?” As this review describes, there is no easy answer because of the many theories and few facts. Nevertheless, we suggest the following, which clinicians can modify to suit their practice and patients. Regarding anatomical/positional changes, it would be possible to say: “There is no simple answer, because the spine is a difficult area to study. It seems likely that the manipulation/adjustment causes some physical changes, but it is not known exactly how. Presently, though, we are fairly confident that the facets, i.e., the small joints at the back of the spine, open up a little bit. There also seems to be a measurable change in the stiffness of the spine immediately after manipulation. We assume that these changes are part of what helps you feel better.”

### Research perspectives

- There has been little research on anatomical mechanisms of SM, and most of the articles we found were not credible according to our assessment methods. The few studies that have been published are on a wide variety of topics, performed by a small number of researchers, and were often small studies (only 7 studies recruited more than 50 participants) that were not followed-up by other similar studies. It seems that there has been no coherent research planning strategy undertaken by any of the manual therapy professions to investigate the anatomical/positional mechanisms of SM. Therefore, there is an opportunity to develop research centres with areas of expertise that can lead high-quality studies in these areas concentrating on anatomically feasible outcome variables.
- Cut points for meaningful changes should be established and should incorporate information such as normal variations, repeatability, and inter-and intra-examiner reliability. The time between the application of SM and the measurement of the potential effect is also important to establish, to infer mechanism(s).
- The results of our review indicate that the 2 most promising areas for further study are changes to facet joint space and spinal stiffness after SM.
- However, after having established what actually happens anatomically in response to SM, it would be important to continue by investigating whether these mechanisms also have a lagged effect and/or result in physiological reactions. Then, this potential chain of events must be linked to the clinical picture, that is, reduction of pain or improvement in function.

## Supplementary Information

Additional file 1.

Additional file 2.

## Data Availability

The datasets used and/or analysed during the current study are available from the corresponding author on reasonable request.
