# Supplementary material for: Mechanisms of manipulation: a systematic review of the literature on immediate anatomical structural or positional changes in response to manually delivered high-velocity, low-amplitude spinal manipulation
Source: Chiropr Man Therap. 2024 Sep 11;32:28. doi: 10.1186/s12998-024-00549-w (PMC11389336; doi:10.1186/s12998-024-00549-w)
Supplement: Supplementary file 1 — Additional file 1. [file 12998_2024_549_MOESM1_ESM.docx]

**ADDITIONAL FILE 2: Exclusion criteria**

- Study type
  - Review articles (systematic reviews, scoping reviews, narrative reviews), guidelines, letters, editorials, commentaries, unpublished manuscripts, dissertations, government reports, books or book chapters, conference proceedings, meeting abstracts, lectures and addresses, diagnostic/measurement tool evaluations, reliability studies, consensus development statements, or guideline statements
- Body areas
  - TMJ, pubic symphysis, hips, extremities
- Outcomes
  - Studies that exclusively reported on clinical outcomes (e.g. decreased pain, improved function) in which no objective anatomical/biomechanical parameters were measured
    - For this study, we consider posture, scoliosis, postural sway, and range of motion to be clinical outcomes and therefore excluded.
    - Pressure pain threshold (PPT) was excluded because patient-reported pain is subjective. Stiffness is objectively measurable so if a tool was used to measure stiffness it was included; if manual palpation was the method, stiffness was excluded due to subjectivity.
- Intervention
  - Studies in which the effect of spinal manipulation could not be isolated from other types of physical interventions for biomechanical outcomes (e.g, studies where spinal manipulation was included in a multimodal program of care, studies where spinal and extremity manipulation were performed on the same cohort of patients)
  - Studies where there was an extended delay in measuring the outcome (e.g. outcomes were only measured after a course of care (days/weeks/months) rather than directly after a manipulation.)
  - Papers investigating cranial and craniosacral methods were excluded as we could find no evidence that they employed HVLA manipulation.
  - Sound created during manipulation was considered physiological, however repositioning of joint surfaces lasting beyond the time of the manipulative procedure was considered anatomical.
- Transient effect only measured
  - Studies that only measured an effect during the manipulation procedure (e.g. force plate analysis) were not included. However, we set no minimum time after the manipulation was completed for the effects to be measured. So, if a paper reported an effect that only lasted a few seconds, it was included.
