# Supplementary material for: Mechanisms of manipulation: a systematic review of the literature on immediate anatomical structural or positional changes in response to manually delivered high-velocity, low-amplitude spinal manipulation
Source: Chiropr Man Therap. 2024 Sep 11;32:28. doi: 10.1186/s12998-024-00549-w (PMC11389336; doi:10.1186/s12998-024-00549-w)
Supplement: Supplementary file 2 — Additional file 2. [file 12998_2024_549_MOESM2_ESM.docx]

**Database: Ovid MEDLINE(R) ALL <1946 to June 05, 2023>**

**Date searched: 06/06/2023**

1 musculoskeletal manipulations/ or kinesiology, applied/ or manipulation, orthopedic/ or manipulation, osteopathic/ or Manipulation, Chiropractic/ or manipulation, spinal/ or motion therapy, continuous passive/

2 ((Manipulati* or adjust*) adj6 (Spine or spinal or Lumbar or Thoracolumbar or Thoracic or Cervico-thoracic or Cervical or Cranial or Atlanto-occipital or Atlantoaxial or Uncovertebral or Zygapophyseal or Facet or Costovertebral or Costotransverse or Rib or Sacroiliac or Sacrococcygeal or medical)).ti,ab,kw,kf.

3 (Chiropract* or osteopath* or naprapath*).ti,ab,kw,kf.

4 ((physiotherap* or ((manual or physical) adj3 therap*)) and manip*).ti,ab,kw,kf.

5 or/1-4

6 exp Spine/

7 rib cage/ or exp ribs/

8 Cranial Sutures/

9 Zygapophyseal Joint/

10 Sacroiliac Joint/

11 Atlanto-Axial Joint/

12 Atlanto-Occipital Joint/

13 (Spine or spinal or Lumbar or Thoracolumbar or Thoracic or Cervico-thoracic or Cervical or Cranial or Atlanto-occipital or Atlantoaxial or Uncovertebral or Zygapophyseal or Facet or Costovertebral or Costotransverse or Rib or Sacroiliac or Sacrococcygeal).ti,ab,kw,kf.

14 or/6-13

15 5 and 14

16 limit 15 to english language

**Database: Embase (Ovid) <1974 to 2023 June 05>**

**Date searched: 06/06/2023**

1 musculoskeletal manipulation/ or kinesiology/ or orthopedic manipulation/ or osteopathic manipulation/ or chiropractic manipulation/ or spine manipulation/ or manipulative medicine/ or movement therapy/

2 ((Manipulati* or adjust*) adj6 (Spine or spinal or back or neck or Lumbar or Thoracolumbar or Thoracic or Cervico-thoracic or cervicothoracic or Cervical or Cranial or Atlanto-occipital or Atlantooccipital or Atlanto-axial or Atlantoaxial or Uncovertebral or Zygapophyseal or Facet or Costovertebral or Costotransverse or Rib or Sacroiliac or Sacrococcygeal or medical)).ti,ab,kw,kf.

3 (Chiropract* or osteopath* or naprapath*).ti,ab,kw,kf.

4 ((physiotherap* or ((manual or physical) adj3 therap*)) and manip*).ti,ab,kw,kf.

5 or/1-4

6 exp spine/

7 exp rib/ or rib cage/

8 cranial suture/

9 zygapophyseal joint/

10 sacroiliac joint/

11 atlantoaxial joint/

12 atlantooccipital joint/

13 (spine or spinal or lumbar or thoracolumbar or thoracic or cervico-thoracic or cervicothoracic or cervical or cranial or atlanto-occipital or atlantooccipital or atlanto-axial or atlantoaxial or uncovertebral or zygapophyseal or facet or costovertebral or costotransverse or rib or sacroiliac or sacrococcygeal).ti,ab,kw,kf.

14 or/6-13

15 5 and 14

16 limit 15 to english language

**Database: CINAHL Ultimate (EBSCOhost)**

**Date searched: 06/06/2023**

S15 S5 AND S13

S14 S5 AND S13

S13 S6 OR S7 OR S8 OR S9 OR S10 OR S11 OR S12

S12 TI ( spine or spinal or lumbar or thoracolumbar or thoracic or "cervico-thoracic" or cervicothoracic or cervical or cranial or "atlanto-occipital" or atlantooccipital or "atlanto-axial" or atlantoaxial or uncovertebral or zygapophyseal or facet or costovertebral or costotransverse or rib or sacroiliac or sacrococcygeal ) OR AB ( spine or spinal or lumbar or thoracolumbar or thoracic or "cervico-thoracic" or cervicothoracic or cervical or cranial or "atlanto-occipital" or atlantooccipital or "atlanto-axial" or atlantoaxial or uncovertebral or zygapophyseal or facet or costovertebral or costotransverse or rib or sacroiliac or sacrococcygeal ) OR SU ( spine or spinal or lumbar or thoracolumbar or thoracic or "cervico-thoracic" or cervicothoracic or cervical or cranial or "atlanto-occipital" or atlantooccipital or "atlanto-axial" or atlantoaxial or uncovertebral or zygapophyseal or facet or costovertebral or costotransverse or rib or sacroiliac or sacrococcygeal )

S11 (MH "Atlanto-Occipital Joint")

S10 (MH "Atlanto-Axial Joint")

S9 (MH "Sacroiliac Joint")

S8 (MH "Zygapophyseal Joint")

S7 (MH "Ribs")

S6 (MH "Spine+")

S5 S1 OR S2 OR S3 OR S4

S4 TI ( ((physiotherap* or ((manual or physical) N3 therap*)) and manip*) ) OR AB ( ((physiotherap* or ((manual or physical) N3 therap*)) and manip*) ) OR SU ( ((physiotherap* or ((manual or physical) N3 therap*)) and manip*) )

S3 TI ( (Chiropract* or osteopath* or naprapath*) ) OR AB ( (Chiropract* or osteopath* or naprapath*) ) OR SU ( (Chiropract* or osteopath* or naprapath*) )

S2 TI ( (Manipulati* or adjust*) N6 (Spine or spinal or back or neck or Lumbar or Thoracolumbar or Thoracic or "Cervico-thoracic" or cervicothoracic or Cervical or Cranial or "Atlanto-occipital" or Atlantooccipital or "Atlanto-axial" or Atlantoaxial or Uncovertebral or Zygapophyseal or Facet or Costovertebral or Costotransverse or Rib or Sacroiliac or Sacrococcygeal or medical) ) OR AB ( (Manipulati* or adjust*) N6 (Spine or spinal or back or neck or Lumbar or Thoracolumbar or Thoracic or "Cervico-thoracic" or cervicothoracic or Cervical or Cranial or "Atlanto-occipital" or Atlantooccipital or "Atlanto-axial" or Atlantoaxial or Uncovertebral or Zygapophyseal or Facet or Costovertebral or Costotransverse or Rib or Sacroiliac or Sacrococcygeal or medical) ) OR SU ( (Manipulati* or adjust*) N6 (Spine or spinal or back or neck or Lumbar or Thoracolumbar or Thoracic or "Cervico-thoracic" or cervicothoracic or Cervical or Cranial or "Atlanto-occipital" or Atlantooccipital or "Atlanto-axial" or Atlantoaxial or Uncovertebral or Zygapophyseal or Facet or Costovertebral or Costotransverse or Rib or Sacroiliac or Sacrococcygeal or medical) )

S1 (MH "Manipulation, Orthopedic") OR (MH "Manipulation, Chiropractic") OR (MH "Manipulation, Osteopathic") OR (MH "Motion Therapy, Continuous Passive") OR (MH "Applied Kinesiology")

**Database: AMED (EBSCOhost)**

**Date searched: 06/06/2023**

S10 S5 AND S8

S9 S5 AND S8

S8 S6 OR S7

S7 TI ( spine or spinal or lumbar or thoracolumbar or thoracic or "cervico-thoracic" or cervicothoracic or cervical or cranial or "atlanto-occipital" or atlantooccipital or "atlanto-axial" or atlantoaxial or uncovertebral or zygapophyseal or facet or costovertebral or costotransverse or rib or sacroiliac or sacrococcygeal ) OR AB ( spine or spinal or lumbar or thoracolumbar or thoracic or "cervico-thoracic" or cervicothoracic or cervical or cranial or "atlanto-occipital" or atlantooccipital or "atlanto-axial" or atlantoaxial or uncovertebral or zygapophyseal or facet or costovertebral or costotransverse or rib or sacroiliac or sacrococcygeal ) OR KW ( spine or spinal or lumbar or thoracolumbar or thoracic or "cervico-thoracic" or cervicothoracic or cervical or cranial or "atlanto-occipital" or atlantooccipital or "atlanto-axial" or atlantoaxial or uncovertebral or zygapophyseal or facet or costovertebral or costotransverse or rib or sacroiliac or sacrococcygeal )

S6 ((((((ZU "spine")) or ((ZU "ribs"))) or ((ZU "cranial sutures"))) or ((ZU "zygapophyseal joint"))) or ((ZU "sacroiliac joint"))) or ((ZU "atlanto-axial joint"))

S5 S1 OR S2 OR S3 OR S4

S4 TI ( ((physiotherap* or ((manual or physical) N3 therap*)) and manip*) ) OR AB ( ((physiotherap* or ((manual or physical) N3 therap*)) and manip*) ) OR KW ( ((physiotherap* or ((manual or physical) N3 therap*)) and manip*) )

S3 TI ( Chiropract* or osteopath* or naprapath* ) OR AB ( Chiropract* or osteopath* or naprapath* ) OR KW ( Chiropract* or osteopath* or naprapath* )

S2 TI ( (Manipulati* or adjust*) N6 (Spine or spinal or back or neck or Lumbar or Thoracolumbar or Thoracic or "Cervico-thoracic" or cervicothoracic or Cervical or Cranial or "Atlanto-occipital" or Atlantooccipital or "Atlanto-axial" or Atlantoaxial or Uncovertebral or Zygapophyseal or Facet or Costovertebral or Costotransverse or Rib or Sacroiliac or Sacrococcygeal or medical) ) OR AB ( (Manipulati* or adjust*) N6 (Spine or spinal or back or neck or Lumbar or Thoracolumbar or Thoracic or "Cervico-thoracic" or cervicothoracic or Cervical or Cranial or "Atlanto-occipital" or Atlantooccipital or "Atlanto-axial" or Atlantoaxial or Uncovertebral or Zygapophyseal or Facet or Costovertebral or Costotransverse or Rib or Sacroiliac or Sacrococcygeal or medical) ) OR KW ( (Manipulati* or adjust*) N6 (Spine or spinal or back or neck or Lumbar or Thoracolumbar or Thoracic or "Cervico-thoracic" or cervicothoracic or Cervical or Cranial or "Atlanto-occipital" or Atlantooccipital or "Atlanto-axial" or Atlantoaxial or Uncovertebral or Zygapophyseal or Facet or Costovertebral or Costotransverse or Rib or Sacroiliac or Sacrococcygeal or medical) )

S1 ((((((ZU "musculoskeletal manipulations")) or ((ZU "applied kinesiology"))) or ((ZU "manipulation orthopedic") or (ZU "manipulation osteopathic") or (ZU "manipulative therapies"))) or ((ZU "manipulation chiropractic"))) or ((ZU "spinal manipulation"))) or ((ZU "motion therapy continuous passive"))

**Database: Cochrane Library all databases (via Wiley)**

**Date searched: 06/06/2023**

#1 MeSH descriptor: [Musculoskeletal Manipulations] this term only

#2 MeSH descriptor: [Kinesiology, Applied] this term only

#3 MeSH descriptor: [Manipulation, Orthopedic] this term only

#4 MeSH descriptor: [Manipulation, Osteopathic] this term only

#5 MeSH descriptor: [Manipulation, Chiropractic] this term only

#6 MeSH descriptor: [Manipulation, Spinal] this term only

#7 MeSH descriptor: [Motion Therapy, Continuous Passive] this term only

#8 ((Manipulati* or adjust*) NEAR/6 (Spine or spinal or back or neck or Lumbar or Thoracolumbar or Thoracic or Cervico-thoracic or cervicothoracic or Cervical or Cranial or Atlanto-occipital or Atlantooccipital or Atlanto-axial or Atlantoaxial or Uncovertebral or Zygapophyseal or Facet or Costovertebral or Costotransverse or Rib or Sacroiliac or Sacrococcygeal or medical)):ti,ab,kw

#9 (Chiropract* or osteopath* or naprapath*):ti,ab,kw

#10 (((physiotherap* or ((manual or physical) NEAR/3 therap*)) and manip*)):ti,ab,kw

#11 {OR #1-#10}

#12 MeSH descriptor: [Spine] explode all trees

#13 MeSH descriptor: [Rib Cage] this term only

#14 MeSH descriptor: [Ribs] explode all trees

#15 MeSH descriptor: [Cranial Sutures] this term only

#16 MeSH descriptor: [Zygapophyseal Joint] this term only

#17 MeSH descriptor: [Sacroiliac Joint] this term only

#18 MeSH descriptor: [Atlanto-Axial Joint] this term only

#19 MeSH descriptor: [Atlanto-Occipital Joint] this term only

#20 (spine or spinal or lumbar or thoracolumbar or thoracic or "cervico-thoracic" or cervicothoracic or cervical or cranial or "atlanto-occipital" or atlantooccipital or "atlanto-axial" or atlantoaxial or uncovertebral or zygapophyseal or facet or costovertebral or costotransverse or rib or sacroiliac or sacrococcygeal):ti,ab,kw

#21 {OR #12-#20}

#22 #11 AND #21

**Database: PEDro (**[**https://pedro.org.au/**](https://pedro.org.au/)**)**

**Date searched: 06/06/2023**

Title and Abstract: spin* AND manipulat*

**Database: Index to Chiropractice Literature (**[**https://www.chiroindex.org/**](https://www.chiroindex.org/)**)**

**Date searched: 06/06/2023**

All Fields:spin* AND All Fields:manipulat*,
